# Characteristics of the Ciliary Body in Healthy Chinese Subjects Evaluated by Radial and Transverse Imaging of Ultrasound Biometric Microscopy

**DOI:** 10.3390/jcm11133696

**Published:** 2022-06-27

**Authors:** Jiawei Ren, Xinbo Gao, Liming Chen, Huishan Lin, Yao Liu, Yuying Zhou, Yunru Liao, Chunzi Xie, Chengguo Zuo, Mingkai Lin

**Affiliations:** 1 State Key Laboratory of Ophthalmology, Guangzhou 510060, China; renjiawei1016@126.com (J.R.); gaoxb@mail.sysu.edu.cn (X.G.); clm222000@163.com (L.C.); linhsh6@mail2.sysu.edu.cn (H.L.); willmakeit@163.com (Y.L.); zhouyuying1220@163.com (Y.Z.); liaoyr5@mail.sysu.edu.cn (Y.L.); 17688806270@163.com (C.X.); 2 Zhongshan Ophthalmic Center, Sun Yat-sen University, Guangzhou 510060, China; 3 Guangdong Provincial Key Laboratory of Ophthalmology and Visual Science, Guangzhou 510060, China

**Keywords:** ciliary body, radial scan, transverse scan, UBM, healthy Chinese subjects

## Abstract

Background: The imaging and analysis of the ciliary body (CB) are valuable in many potential clinical applications. This study aims to demonstrate the anatomy characteristics of CB using radial and transverse imaging of ultrasound biometric microscopy (UBM) in healthy Chinese subjects, and to explore the determining factors. Methods: Fifty-four eyes of 30 healthy Chinese subjects were evaluated. Clinical data, including age, body mass index (BMI), intraocular pressure (IOP), axial length (AL), and lens thickness (LT), were collected. Radial and transverse UBM measurements of the ciliary body were performed. Anterior chamber depth (ACD), ciliary sulcus diameter (CSD), ciliary process length (CPL), ciliary process density (CPD), ciliary process area (CPA), ciliary muscle area (CMA), ciliary body area (CBA), ciliary body thickness (CBT_0_, CBT_1_, and CBT_max_), anterior placement of ciliary body (APCB), and trabecular-ciliary angle (TCA) of four (superior, nasal, inferior, and temporal) quadrants were measured. Results: The average CPL was 0.513 ± 0.074 mm, and the average CPA was 0.890 ± 0.141 mm^2^. CPL and CPA tended to be longer and larger in the superior quadrant (*p* < 0.001) than in the other three quadrants. Average CPL was significantly correlated with AL (r = 0.535, *p* < 0.001), ACD (r = 0.511, *p* < 0.001), and LT (r = −0.512, *p* < 0.001). Intraclass correlation coefficient (ICC) scores were high for CPL (0.979), CPD (0.992), CPA (0.966), CMA (0.963), and CBA (0.951). Conclusions: In healthy Chinese subjects, CPL was greatest in the superior quadrant, followed by the inferior, temporal, and nasal quadrants, and CPA was largest in the superior quadrant, followed by the tempdoral, inferior, and nasal quadrants. Transverse UBM images can be used to measure the anatomy of the ciliary process with relatively good repeatability and reliability.

## 1. Introduction

The ciliary body (CB) is the middle part of the anterior uvea. Anatomically, the CB spans the portion of the eye between the scleral spur and the ora serrata. In the sagittal section, the CB is triangular and divided into two parts: the pars plicata, characterized by a longitudinal radial process called the ciliary process (CP), and the pars plana, which is flat and approximately 4 mm behind the CP [1]. The CB has an important relationship with neighboring structures and has a variety of functions, including aqueous humor production, regulation of aqueous humor output through the uveal sclera pathway, and regulation via the ciliary muscle and suspensory ligament [2]. However, the CB and CP cannot be directly visualized due to the posterior position.

Ultrasound biometric microscopy (UBM) has been widely used to analyze anterior chamber structures, especially the structure behind the iris, such as the CB [3,4]. The UBM imaging and analysis of the CB, based on the radial scan, is valuable in many potential clinical applications, such as analyzing the pathogenesis of different types of angle-closure glaucoma [5,6,7,8]; describing the effects of pharmacologic agents on CB [9,10,11]; revealing the relationship between CBT and refractive error [12]; and assessing the development of uveitis, tumors, and cysts [13,14,15]. 

In radial UBM images, only one CB can be seen at one time, and only the pars plana and plicata of the CB can be visualized. In addition, it cannot clearly characterize the morphological features of the CPs due to their irregular arrangement, similar to protuberance. However, a transverse scan of CB is available by UBM. In transverse images, the transducer probe can be aligned with an entire row of CPs to illustrate the complex anatomy of the CB. Such images provide the ability to analyze a group of CPs rather than a single CP as in a radial scan. However, as far as we know, an objective and repeatable protocol of transverse scanning of UBM has never been reported. In this study, we aim to demonstrate the anatomy characteristics of the ciliary body using both radial and transverse scans of UBM in healthy Chinese subjects, and to explore the determining factors for further study of the pathogenesis, prevention, and follow-up of CB-related disease in vivo.

## 2. Materials and Methods

### 2.1. Participants

This was a cross-sectional study consisting of healthy Chinese subjects. The research conformed to the Helsinki Declaration’s guidelines; was approved by the Ethics Board of the Zhongshan Ophthalmic Center (ZOC), Sun Yat-Sen University; and participants signed informed consents. All subjects underwent detailed ocular examinations, including slit-lamp examination, fundus examination with a 90-diopter lens, and intraocular pressure (IOP) measurement by Goldmann applanation tonometry. Axial length (AL), lens thickness (LT), white-to-white (WTW) corneal diameter, and central corneal thickness (CCT) were measured by the same trained observer (X.G.) via IOL Master 700 (Carl Zeiss Meditec AG, Jena, Germany, version 1.7). High-definition images of the anterior segment and the CB structures were provided by UBM.

The inclusion criteria were: (1) Chinese ethnicity, (2) age ≥ 18 years, (3) IOP < 21 mmHg by Goldmann applanation tonometry, and (4) normal optic disc and macular appearance. The exclusion criteria were: (1) any intraocular disease except moderate cataracts, (2) history of ocular trauma, (3) history of eye surgery, (4) ocular surface active inflammation, (5) refractive error exceeding 5.00 diopters of hyperopia/myopia or 2.00 diopters of astigmatism, and (6) systemic disorders that affect visual functions.

### 2.2. Image Acquisition

UBM imaging was performed using an sw-3200L UBM and 50 MHz linear transducer (Tianjin Suowei Electronic Technology Co., Ltd., Tianjin, China). The highest axial and lateral resolutions of the UBM were no less than 40 μm. All images included in this study were obtained by the same examiner (L.C.). Radial scans were performed in the positions of 9, 12, 3, and 6 o’clock centered over the corneal limbus, and perpendicular sulcus-to-sulcus scans were obtained over the pupil center.

**Figure 1:**
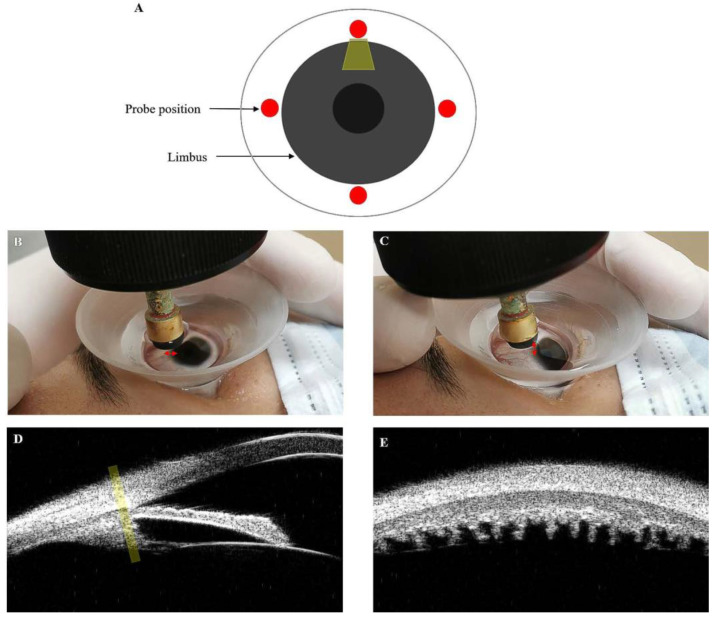
UBM was used to obtain the images of the ciliary body. The ultrasound beam appears in yellow. (**A**) Enface view with the probe position at 9, 12, 3, and 6 o’clock perpendicular to the limbus in red. (**B**,**C**) These images show the position of probes and eyes when obtaining superior radial and transverse UBM images, respectively. The red arrow shows the direction in which the probe swings during the examination. (**D**) This UBM image shows the relationship of the ultrasound beam to the CB when the transverse scan is performed. (**E**) This image depicts a transverse (superior) UBM image.

Specifically, transverse scans of UBM images were obtained as follows. The probe was perpendicular to the corneal limbus, and the first and most precise CPs image at the time of disappearance of the ciliary sulcus was obtained from four different quadrants (superior, nasal, inferior, and temporal) of each eye. Measurements of the superior quadrant were performed repeatedly over for 1 h by the same physician while masking the initial results. The images were analyzed by the same observer. Figure 1A–D show the probe directions. Figure 1E shows a transverse (superior) CPs image sample. 

### 2.3. Image Measurement

**Figure 2:**
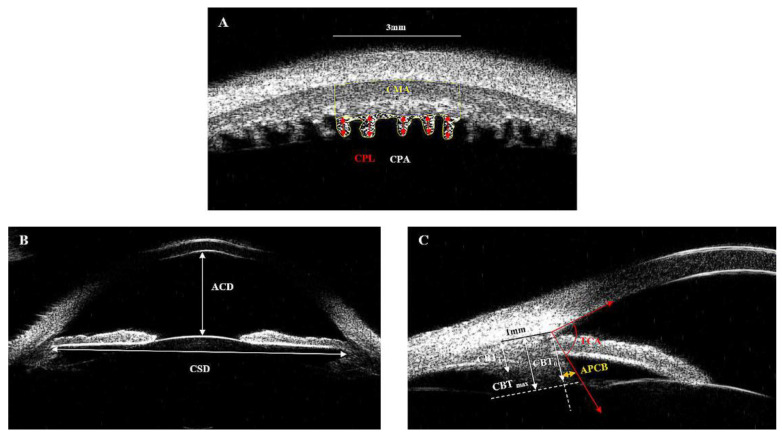
Measurement of ciliary parameters in UBM images. (**A**) Within the boundaries of CPs and a 3-mm linear distance, the ciliary process length (CPL) was measured by calculating the average length of each individual CP. Each individual CPL is shown by the red arrow. The CPD was defined as the number of ciliary processes. The CPA, shown as the shaded area, was measured by calculating the area of ciliary processes. The CMA, the area above the CPA, circled by yellow lines, was measured by calculating the area of the ciliary muscle. The CBA was the sum area of the CPA and the CMA. (**B**) ACD, anterior chamber depth; CSD, ciliary sulcus diameter. (**C**) CBT_0_, ciliary body thickness at the point of the scleral spur; CBT_1_, ciliary body thickness at a distance of 1 mm from the scleral spur; CBT_max_, maximum ciliary body thickness; APCB, anterior placement of the ciliary body; TCA, trabecular-ciliary angle.

As Figure 2A shows: (1) the ciliary process length (CPL) was determined by calculating the average length of each individual CP within a 3-mm line in a row; (2) the ciliary process density (CPD) was defined as the overall number of ciliary processes in a 3-mm segment of the transverse CB; (3) the ciliary process area (CPA), ciliary muscle area (CMA), and ciliary body area (CBA) were measured by calculating the area of ciliary processes, ciliary muscle, and CB, respectively, calculating the area within the boundaries of CPs within a 3-mm linear distance using ImageJ software 1.51 (ImageJ Software Inc., Bethesda, MD, USA). The method and the parameters, such as CPL, CPA, CPD, CMA, and CBA of transverse UBM scans, were first defined in this study, so we analyzed the intra-observer reproducibility.

As Figure 2B shows: anterior chamber depth (ACD) was defined as the axial distance between the corneal endothelium and the anterior lens surface. Ciliary sulcus diameter (CSD) was the perpendicular sulcus-to-sulcus distance from 12 to 6 o’clock.

As Figure 2C shows: (1) CBT_0_ was the CBT at the point of the scleral spur, and CBT_1_ was the CBT at a distance of 1 mm from the scleral spur; (2) maximum CBT (CBT_max_) was defined as the distance between the innermost point of the CB and the inner surface of the sclera; (3) anterior placement of the ciliary body (APCB) was the distance from the most anterior point of the CB to the vertical line drawn from the inner surface of the scleral spur; (4) the trabecular-ciliary angle (TCA) refers to the angle between the posterior corneal surface and the anterior surface of the CB.

### 2.4. Statistical Analysis

All data were imported and sorted by two authors. The statistical analysis and description were performed in SPSS 23 (IBM Corporation, Chicago, IL, USA). One-way analysis of variance (ANOVA) was performed to compare the CPL, CPD, CPA, CMA, CBA, CBT, and APCB in each of the four quadrants. The CPL, CPD, CPA, CMA, CBA, CBT, and APCB values obtained in the four quadrants were averaged, and the Pearson’s correlation coefficient test was used to assess the relationships of average CPL, CPD, CPA, CMA, CBA, CBT, and APCB with the following parameters: age, BMI, AL, ACD, LT, and CSD. The intraclass correlation coefficient (ICC) and the Bland–Altman plots were used to analyze the consistency of each parameter for two repeat examinations of the superior CP. *p* < 0.05 was considered to be statistically significant.

## 3. Results

### 3.1. Patient Characteristics

Data for this study consisted of 54 eyes of 30 healthy adults. The mean age of the participants was 38.07 ± 12.58 years (range: 18–65 years). Subjects included 14 females and 16 males, with an average BMI of 23.27 ± 3.14 (range: 17.67 to 29.27). Table 1 shows that the mean IOP, CSD, AL, ACD, LT, WTW, and CCT were 14.59 ± 2.13 mmHg, 11.56 ± 0.53 mm, 24.46 ± 1.09 mm, 3.41 ± 0.30 mm, 3.93 ± 0.37 mm, 11.99 ± 0.30 mm, and 528.84 ± 29.81 mm, respectively.

### 3.2. Intra-Observer Reproducibility of the Parameters in Transverse UBM Scans of CB

**Figure 3:**
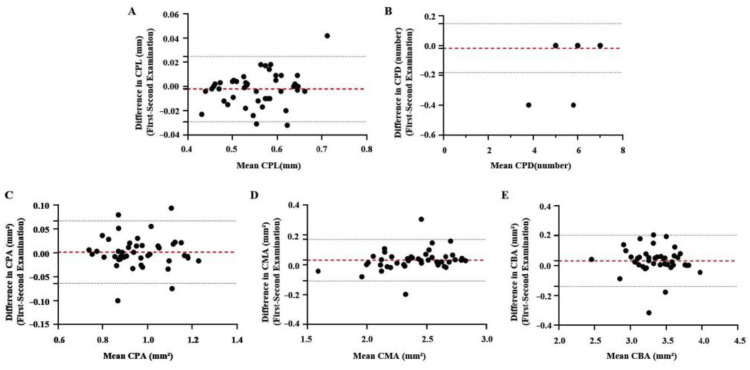
Bland–Altman plots for two repeat examinations of the superior CP. Differences for CPL (**A**), CPD (**B**), CPA (**C**), CMA (**D**), and CBA (**E**) generally fell within the repeatability coefficient limit (black dashed line), suggesting that the mean parameter was generally repeatable. The red dashed line shows the mean.

Intraclass correlation coefficient (ICC) scores describe the level of absolute agreement between two examinations and provide a measure of reproducibility. In our study, there were 44 images in total. ICC scores were high for CPL (0.979), CPD (0.992), CPA (0.966), CMA (0.963), and CBA (0.951) (Table 2). For all parameters measured, there was good agreement between the two examinations. The mean difference and 95% limits of agreement (LoA) in CPL, CPD, CPA, CMA, and CBA between the first and second examinations were −0.002 (−0.029, 0.024), −0.018 (−0.183, 0.147), 0.001 (−0.064, 0.066), 0.030 (−0.108, 0.169), and 0.031 (−0.140, 0.203), respectively. Differences were plotted against the mean, as shown by the Bland–Altman plots in Figure 3.

### 3.3. Anatomy Characteristics of the CB Both from Radial and Transverse Imaging of UBM

Table 3 showed that the average CPL was 0.513 ± 0.074 mm, the average CPA was 0.890 ± 0.141 mm^2^, and the average CMA was 2.381 ± 0.280 mm^2^ in the four quadrants. The CPL was 0.558 ± 0.070 mm, 0.490 ± 0.062 mm, 0.505 ± 0.075 mm, and 0.498 ± 0.072 mm in the superior, inferior, temporal, and nasal quadrants, respectively. The CPA was 0.964 ± 0.130 mm^2^, 0.841 ± 0.112 mm^2^, 0.865 ± 0.142 mm^2^, and 0.889 ± 0.148 mm^2^, whereas the CMA was 2.327 ± 0.312 mm^2^, 2.483 ± 0.245 mm^2^, 2.355 ± 0.270 mm^2^, and 2.361 ± 0.271 mm^2^, respectively. There were significant differences in the CPL (*p* < 0.001), CPA (*p* < 0.001), and CMA (*p* = 0.019) among the four different quadrants (Table 3). However, no statistically significant differences in the average CPD, CBA, CBT_0_, CBT_1_, CBT_max_, APCB, and TCA (*p* > 0.05) among the four different quadrants were found.

**Figure 4:**
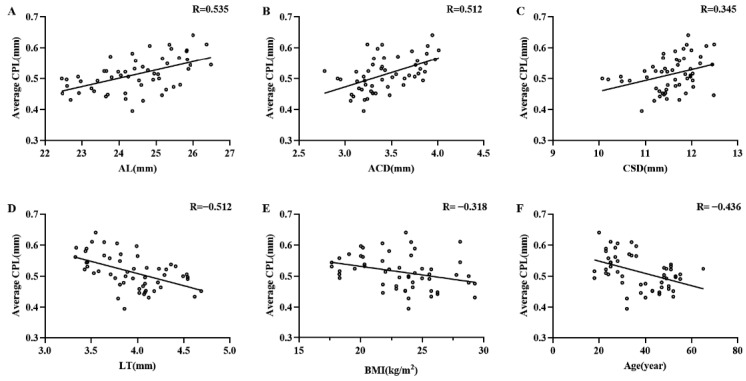
Scatter plots of AL (**A**), ACD (**B**), CSD (**C**), LT (**D**), BMI (**E**), age (**F**), and average CPL in all recruited eyes. The longer the AL, the deeper the ACD, and the larger CSD were, the longer the CPL was. The smaller the BMI and the thinner the LT were, the longer the CPL was. The younger the subject was, the longer the CPL was. The solid line represents the best-fit line.

The correlations between average CPL, CPD, CPA, CMA, CBA, CBT_0_, CBT1, CBT_max_, APCB, TCA, and various other clinical parameters are shown in Table 4. Scatter plots of average CPL and other ocular and systemic parameters are shown in Figure 4. Average CPL was significantly correlated with age (r = −0.436, *p* = 0.001), BMI (r = −0.318, *p* = 0.019), AL (r = 0.535, *p* *<* 0.001), ACD (r = 0.512, *p* *<* 0.001), LT (r = −0.512, *p* *<* 0.001), and CSD (r = 0.345, *p* = 0.011). Average CPD was positively correlated with age (r = 0.354, *p* = 0.013) and LT (r = 0.421, *p* = 0.002), and negatively correlated with AL (r = −0.445, *p* = 0.001), ACD (r = −0.343, *p* = 0.009), and CSD (r = −0.375, *p* = 0.005). There was no significant correlation between the average CPA and other clinical parameters (*p* > 0.05) or the CBT_max_. Average CMA showed a significant correlation with age (r = 0.488, *p* *<* 0.001), BMI (r = 0.349, *p* = 0.010), AL (r =−0.279, *p* = 0.041), and LT (r = 0.519, *p* *<* 0.001), but not with ACD and CSD (*p* > 0.05). Average CBA was not related to AL, ACD, or CSD in all eyes (*p* > 0.05), except for a significant positive correlation with age (r = 0.439, *p* *<* 0.001), BMI (r = 0.307, *p* = 0.024), and LT (r = 0.432, *p* *<* 0.001). Average CBT_0_ showed a significant correlation with LT (r = 0.357, *p* = 0.008), but not with age, BMI, AL, ACD, or CSD (*p* > 0.05). There was only a statistically significant correlation between the average CBT_1_ and CSD (r = 0.287, *p* = 0.035). Average APCB was significantly correlated with age (r = 0.483, *p <* 0.001), AL (R = −0.513, *p <* 0.001), ACD (r = −0.542, *p <* 0.001), LT (r = 0.491, *p <* 0.001), and CSD (r = −0.566, *p <* 0.001), but not with BMI (*p* > 0.005). There was a significant correlation between average TCA and age (r = −0.512, *p* *<* 0.001), BMI (r = −0.352, *p* = 0.009), AL (r = 0.464, *p* *<* 0.001), ACD (r = 0.649, *p* *<* 0.001), LT (r = −0.508, *p* *<* 0.001), and CSD (r = 0.569, *p* *<* 0.001).

## 4. Discussion

The study of CB has gained significant attention because of its involvement in glaucoma and myopia. Several studies have been conducted on humans to provide histological information about the CB, but all have encountered postmortem shrinkage problems [16,17]. UBM can be used to measure any quadrant on living subjects, as it is unaffected by shrinkage. Since its inception, UBM has been used in many clinical and preclinical studies associated with CB-related disease, and it is irreplaceable for the study of the posterior chamber and the innermost structures of the iridociliary region compared to AS-OCT [4,18,19,20]. To the best of our knowledge, this is the first report that demonstrated the morphology parameters of the CB from both radial and transverse scans in four different quadrants in vivo of Chinese people and the correlations between them with systemic and ocular parameters.

In our study, four quadrantal analyses of the ciliary process revealed that the CPL was longest in the superior quadrant, followed by the inferior, temporal, and nasal quadrants. CPA tended to be larger in the superior quadrant than in the other three quadrants. In contrast to CPL, CMA was largest in the nasal quadrant, followed by the temporal, inferior, and superior quadrants. This may be due to the embryonic development of the eye. Numerous reports about ocular morphology have been published. Retinal nerve fiber layer (RNFL) thickness was found to be thicker in the superior and inferior quadrants, followed by the temporal and nasal quadrants in normal Chinese students aged 6 to 17 years using optical coherence tomography (OCT) [21]. Corneal thickness was lower in the inferotemporal quadrant and higher in the superonasal quadrant using anterior segmental-optical coherence tomography (AS-OCT) [22]. A previous study compared the ciliary body morphology between Caucasians and Chinese individuals aged 40 to 80 years, and found that Chinese individuals had a thinner CBT using UBM [23]. It was reported that CBT_1_ was significantly thicker in the superior quadrant than in the nasal, temporal, and inferior quadrants using UBM in Asian subjects aged 11–86 years [24]. However, in our study, no significant difference in CBT_0_, CBT_1_, and CBT_max_ among the four quadrants was found. The inconsistency may arise from the inclusion of subjects of different races. APCB and TCA were inversely related to anterior rotation of the ciliary body. According to a previous study in Japan, the mean TCA was 79.2 degrees using UBM [3], which is similar to our findings.

Our study provided a new protocol to scan transverse (quadrant) CP images using UBM; in particular, a method to locate the anatomical point to achieve a repeatable fashion. For the first time, we clearly defined CPL as the distance parallel to the long axis of the CP from the farthest end to the midpoint of the line of the bilateral ciliary sulcus according to the morphology. CPD, CPA, CMA, and CBA were all measured within the middle 3-mm linear distance of the transverse CB image. Due to the spherical shape of the eyeball, the surrounding CPs appear deformed in the image at a greater distance. Additionally, if it is at a shorter distance, the number of CPs will be too small, resulting in statistical errors. Therefore, the CPs in the area we chose were the most precise and closest to the actual shape in the scan. To date, there has been no standardized method for reliable measurement of the ciliary process. Only one study published in 2021 obtained CP images of normal human eyes from the temporal pars plicata using UBM, and the ICC values were >0.8 for CP parameters (CPL, CPD, CPA) [25]. However, there was an advantage that all five parameters (CPL, CPD, CPA, CMA, CBA) in our study had ICC values > 0.9, suggesting relatively better repeatability and reliability than the study mentioned above. The reason for this might be that we obtained CP images in our study by focusing the ultrasonic probe perpendicular to the corneal limbus at the time of disappearance of the ciliary sulcus, and this might prove a more reliable method to measure CPs from transverse UBM images.

Additionally, the correlations between parameters of the CP and systemic and ocular parameters in healthy Chinese subjects were revealed, which has not been reported before, as far as we know. In addition, average CPL had a negative correlation with age and BMI, indicating that people who were younger or with lower BMI tended to have a longer CP. In our study, average CPL and average TCA showed a positive correlation with AL and CSD, and showed a negative correlation with LT; average CPD, average CMA, and average APCB showed the opposite correlation. In other words, healthy Chinese subjects, who had smaller eyes with shorter AL, shorter CSD, and thicker lenses, had shorter but denser ciliary processes, thicker ciliary muscle, and more anteriorly located CB, which might provide new inspiration for the occurrence and mechanism of malignant glaucoma in patients with primary angle-closure (PAC) and primary angle-closure glaucoma (PACG). With transverse direction UBM scans, CP can be seen more clearly, which may provide a more accurate way to explore the pathogenesis, and evaluate the surgical prognosis of glaucoma.

In previous studies, CBT_1_ was positively correlated with AL in normal subjects [24], and CBT_2_ and CBT_3_ (2 mm and 3 mm posterior to the scleral spur) were positively correlated with AL in myopic eyes [12]. It was reported that ciliary muscle thickness (CMT_2_ and CMT_3_, 2 mm and 3 mm posterior to the scleral spur) was increased in myopic eyes compared with nonmyopic eyes, suggesting that a large CB may be associated with a greater contraction of the ciliary muscles to resist equatorial sclera thinning [26,27]. However, a correlation between CBT_0_, CBT_1_, CBT_max_, and AL was not found in our study, probably because we used different measured parameters, and the difference was not significant.

As the site of aqueous humor production, the CP is also the site of surgical interventions, such as trans-scleral cyclo-photocoagulation (TSCPC), endoscopic cyclophotocoagulation (ECP), and ultrasound cycloplasty (UCP), which are aimed at destroying the CB to reduce IOP in glaucoma patients [28,29,30]. Therefore, images of the ciliary process using UBM could provide a more refined observation method to evaluate the morphological changes of the ciliary process in TSCPC-, ECP-, and UCP-treated glaucoma and the relationship between the morphological changes and IOP reduction. The ability to study drug or surgery action on the morphology of the CP itself may offer valuable insights regarding the mechanisms of action. 

The main limitation of our study is that the resolution limitations of the UBM technique may have contributed to morphometric errors. The spatial resolution of the UBM is superior in the radial direction compared to the transverse direction. The examination was performed to apply the probe as vertically to the corneal limbus as possible to obtain a precise image. Another significant limitation is the small sample size. Therefore, we need to compensate for the lack of accuracy caused by these limitations by increasing the number of subjects. The third limitation is that we only included Chinese subjects in this study, which limited the generalizability of the results. In order to verify the generalizability of the results, further multiracial studies are warranted to confirm our findings. In addition, even though it does not affect the clinical conclusions, the analysis could be more accurate if the inter-eye correlation was considered.

## 5. Conclusions

In conclusion, we found a topographical distribution of the ciliary body in healthy Chinese subjects using the application of radial and transverse UBM imaging, and provided more information on the ciliary anatomy. The average CPL and CPA were greatest in the superior quadrant. Given the relatively good repeatability and reliability of ciliary process imaging, further studies should be performed to explore the pathogenesis of CB-related diseases, such as malignant glaucoma, and to evaluate the role of glaucoma interventions aimed at the CB, such as TSCPC, ECP, and UCP.

## Figures and Tables

**Table 1:** Demographic, clinical examination, and ocular biometric parameters of subjects.

Variables	Mean ± SD	Median	Range
Age(years)	38.07 ± 12.58	39	18–65
Female gender%	53.33%	-	-
BMI (kg/m^2^)	23.27 ± 3.14	23.27	17.67–29.27
IOP (mmHg)	14.59 ± 2.13	14.45	10.00–19.70
CSD (mm)	11.56 ± 0.53	11.64	10.07–12.49
AL (mm)	24.46 ± 1.09	24.50	22.45–26.49
ACD (mm)	3.44 ± 0.30	3.40	2.78–4.01
LT (mm)	3.93 ± 0.37	3.96	3.33–4.69
WTW (mm)	11.99 ± 0.30	12.00	11.40–12.70
CCT (μm)	528.84 ± 29.81	527.00	478.00–579.00

SD, standard deviation; BMI, body mass index; IOP, intraocular pressure; CSD, ciliary sulcus diameter; AL axial length; ACD, anterior chamber depth; LT, lens thickness; WTW, white-to-white; CCT, central corneal thickness.

**Table 2:** ICC and 95% LoA results for ciliary body anatomy measured in the superior quadrant from the transverse scan.

Measurement	Mean ± SD		ICC, 95%CI	*p*	95%LoA	No. of Images
	Examination 1	Examination 2				
CPL (mm)	0.553 ± 0.069	0.555 ± 0.065	0.979(0.962,0.988)	<0.001	−0.029, 0.024	44
CPD (number)	5.641 ± 0.669	5.660 ± 0.645	0.992(0.985,0.995)	<0.001	−0.183, 0.147	44
CPA (mm^2^)	0.964 ± 0.127	0.962 ± 0.128	0.966(0.939,0.982)	<0.001	−0.064, 0.066	44
CMA (mm^2^)	2.399 ± 0.288	2.368 ± 0.268	0.963(0.923,0.981)	<0.001	−0.108, 0.169	44
CBA (mm^2^)	3.362 ± 0.291	3.333 ± 0.297	0.951(0.907,0.974)	<0.001	−0.140, 0.203	44
CBA (mm^2^)	3.362 ± 0.291	3.333 ± 0.297	0.951(0.907,0.974)	<0.001	−0.140, 0.203	44

*p* < 0.05, F test of ICC with true value 0; SD, standard deviation; ICC, intraclass correlation coefficient; CI, confidence interval; LoA, limits of agreement; CPL, ciliary process length; CPD, ciliary process density (in 3-mm linear distance); CPA, ciliary process area; CMA, ciliary muscle area; CBA, ciliary body area.

**Table 3:** Ciliary body and ciliary process parameters measured in the four quadrants.

Quadrant	Average	Superior	Nasal	Inferior	Temporal	*p*-Value
CPL (mm)	0.513 ± 0.074	0.558 ± 0.070	0.490 ± 0.062	0.505 ± 0.075	0.498 ± 0.072	<0.001
CPD (number)	5.779 ± 0.832	5.596 ± 0.702	5.752 ± 0.790	5.800 ± 0.937	5.969 ± 0.863	0.139
CPA (mm^2^)	0.890 ± 0.141	0.964 ± 0.130	0.841 ± 0.112	0.865 ± 0.142	0.889 ± 0.148	<0.001
CMA (mm^2^)	2.381 ± 0.280	2.327 ± 0.312	2.483 ± 0.245	2.355 ± 0.270	2.361 ± 0.271	0.019
CBA (mm^2^)	3.271 ± 0.292	3.291 ± 0.328	3.323 ± 0.270	3.220 ± 0.282	3.250 ± 0.282	0.272
CBT_0_ (mm)	1.053 ± 0.188	1.014 ± 0.175	1.038 ± 0.142	1.054 ± 0.218	1.104 ± 0.203	0.084
CBT_1_ (mm)	0.811 ± 0.159	0.849 ± 0.181	0.789 ± 0.124	0.798 ± 0.147	0.811 ± 0.173	0.217
CBT_max_ (mm)	1.248 ± 0.169	1.224 ± 0.153	1.214 ± 0.152	1.261 ± 0.177	1.291 ± 0.183	0.064
APCB (mm)	0.349 ± 0.314	0.322 ± 0.144	0.324 ± 0.138	0.358 ± 0.235	0.392 ± 0.214	0.174
TCA (degree)	79.379 ± 10.020	77.859 ± 8.949	80.635 ± 8.587	80.583 ± 10.684	78.437 ± 11.531	0.343

Values are presented as the mean ± standard deviation; *p* < 0.05, one-way analysis of variance; CPL, ciliary process length; CPD, ciliary process density (in 3-mm linear distance); CPA, ciliary process area; CMA, ciliary muscle area; CBA, ciliary body area; CBT_0_, ciliary body thickness at the point of the scleral spur; CBT_1_, ciliary body thickness at a distance of 1 mm from the scleral spur; CBT_max_, maximum ciliary body thickness; APCB, anterior placement of ciliary body; TCA, trabecular-ciliary angle.

**Table 4:** Correlations between ciliary process, CB parameters, and systemic and ocular parameters.

Variables	Average CPL (mm)		Average CPD (Number)		Average CPA (mm^2^)		Average CMA (mm^2^)		Average CBA (mm^2^)		Average CBT_0_ (mm)		Average CBT_1_ (mm)		Average CBT_max_ (mm)		Average APCB (mm)		Average TCA (Degree)	
	r	*p*	r	*p*	r	*p*	r	*p*	r	*p*	r	*p*	r	*p*	r	*p*	r	*p*	r	*p*
Age	−0.436	0.001	0.354	0.013	−0.020	0.884	0.488	<0.001	0.439	<0.001	0.261	0.057	−0.022	0.874	0.154	0.266	0.483	<0.001	−0.512	<0.001
BMI (kg/m^2^)	−0.318	0.019	0.160	0.246	−0.031	0.823	0.349	0.010	0.307	0.024	0.134	0.335	0.130	0.348	−0.011	0.939	0.229	0.096	−0.352	0.009
AL (mm)	0.535	<0.001	−0.445	0.001	0.202	0.143	−0.279	0.041	−0.170	0.218	−0.074	0.595	0.082	0.554	−0.118	0.396	−0.513	<0.001	0.464	<0.001
ACD (mm)	0.512	<0.001	−0.343	0.011	0.192	0.165	−0.336	0.013	−0.227	0.099	−0.195	0.159	0.126	0.364	−0.076	0.587	−0.542	<0.001	0.649	<0.001
LT (mm)	−0.512	<0.001	0.421	0.002	−0.105	0.450	0.519	<0.001	0.432	<0.001	0.357	0.008	0.081	0.560	0.237	0.085	0.491	<0.001	−0.508	<0.001
CSD (mm)	0.345	0.011	−0.375	0.005	−0.013	0.923	−0.275	0.044	−0.257	0.060	−0.022	0.873	0.287	0.035	0.008	0.954	−0.566	<0.001	0.569	<0.001

*p* < 0.05, Pearson’s correlation coefficient. CPL, ciliary process length; CPD, ciliary process density (number in 3-mm linear distance); CPA, ciliary process area; CMA, ciliary muscle area; CBA, ciliary body area; CBT_0_, ciliary body thickness at the point of the scleral spur; CBT_1_, ciliary body thickness at the distance of 1 mm from scleral spur; CBT_max_, maximum ciliary body thickness; APCB, anterior placement of ciliary body; TCA, trabecular-ciliary angle; BMI, body mass index; AL, axial length; ACD, anterior chamber depth; LT, lens thickness; CSD, ciliary sulcus diameter.

## Data Availability

The datasets used and/or analyzed during the current study are available from the corresponding author upon reasonable request.

## References

[1] Kels B.D., Grzybowski A., Grant-Kels J.M. (2015). Human ocular anatomy. Clin. Dermatol..

[2] Coca-Prados M., Escribano J. (2007). New perspectives in aqueous humor secretion and in glaucoma: The ciliary body as a multifunctional neuroendocrine gland. Prog. Retin. Eye Res..

[3] Henzan I.M., Tomidokoro A., Uejo C., Sakai H., Sawaguchi S., Iwase A., Araie M. (2010). Ultrasound biomicroscopic configurations of the anterior ocular segment in a population-based study the Kumejima Study. Ophthalmology.

[4] Fernández-Vigo J.I., Kudsieh B., Shi H., De-Pablo-Gómez-de-Liaño L., Fernández-Vigo J., García-Feijóo J. (2022). Diagnostic imaging of the ciliary body: Technologies, outcomes, and future perspectives. Eur. J. Ophthalmol..

[5] Marchini G., Pagliarusco A., Toscano A., Tosi R., Brunelli C., Bonomi L. (1998). Ultrasound biomicroscopic and conventional ultrasonographic study of ocular dimensions in primary angle-closure glaucoma. Ophthalmology.

[6] Wang Z., Chung C., Lin J., Xu J., Huang J. (2016). Quantitative Measurements of the Ciliary Body in Eyes With Acute Primary-Angle Closure. Investig. Ophthalmol. Vis. Sci..

[7] Wang F., Wang D., Wang L. (2019). Characteristic Manifestations regarding Ultrasound Biomicroscopy Morphological Data in the Diagnosis of Acute Angle Closure Secondary to Lens Subluxation. BioMed Res. Int..

[8] Wang Z., Huang J., Lin J., Liang X., Cai X., Ge J. (2014). Quantitative measurements of the ciliary body in eyes with malignant glaucoma after trabeculectomy using ultrasound biomicroscopy. Ophthalmology.

[9] Alibet Y., Levytska G., Umanets N., Pasyechnikova N., Henrich P.B. (2017). Ciliary body thickness changes after preoperative anti-inflammatory treatment in rhegmatogenous retinal detachment complicated by choroidal detachment. Graefe’s Arch. Clin. Exp. Ophthalmol..

[10] Mishima H.K., Shoge K., Takamatsu M., Kiuchi Y., Tanaka J. (1996). Ultrasound biomicroscopic study of ciliary body thickness after topical application of pharmacologic agents. Am. J. Ophthalmol..

[11] Arakawa A., Tamai M. (2000). Ultrasound biomicroscopic analysis of the human ciliary body after 1 and 2% pilocarpine instillation. Ophthalmologica.

[12] Oliveira C., Tello C., Liebmann J.M., Ritch R. (2005). Ciliary body thickness increases with increasing axial myopia. Am. J. Ophthalmol..

[13] Gentile R.C., Liebmann J.M., Tello C., Stegman Z., Weissman S.S., Ritch R. (1996). Ciliary body enlargement and cyst formation in uveitis. Br. J. Ophthalmol..

[14] Weisbrod D.J., Pavlin C.J., Emara K., Mandell M.A., McWhae J., Simpson E.R. (2006). Small ciliary body tumors: Ultrasound biomicroscopic assessment and follow-up of 42 patients. Am. J. Ophthalmol..

[15] Mannino G., Malagola R., Abdolrahimzadeh S., Villani G.M., Recupero S.M. (2001). Ultrasound biomicroscopy of the peripheral retina and the ciliary body in degenerative retinoschisis associated with pars plana cysts. Br. J. Ophthalmol..

[16] Hara K., Lütjen-Drecoll E., Prestele H., Rohen J.W. (1977). Structural differences between regions of the ciliary body in primates. Investig. Ophthalmol. Vis. Sci..

[17] Tamm E.R., Lütjen-Drecoll E. (1996). Ciliary body. Microsc. Res. Tech..

[18] Dada T., Gadia R., Sharma A., Ichhpujani P., Bali S.J., Bhartiya S., Panda A. (2011). Ultrasound biomicroscopy in glaucoma. Surv. Ophthalmol..

[19] Smith S.D., Singh K., Lin S.C., Chen P.P., Chen T.C., Francis B.A., Jampel H.D. (2013). Evaluation of the anterior chamber angle in glaucoma: A report by the american academy of ophthalmology. Ophthalmology.

[20] Janssens R., van Rijn L.J., Eggink C.A., Jansonius N.M., Janssen S.F. (2021). Ultrasound biomicroscopy of the anterior segment in patients with primary congenital glaucoma: A review of the literature. Acta Ophthalmol..

[21] Chen L., Huang J., Zou H., Xue W., Ma Y., He X., Lu L., Zhu J. (2013). Retinal nerve fiber layer thickness in normal Chinese students aged 6 to 17 years. Investig. Ophthalmol. Vis. Sci..

[22] Li Y., Tan O., Brass R., Weiss J.L., Huang D. (2012). Corneal epithelial thickness mapping by Fourier-domain optical coherence tomography in normal and keratoconic eyes. Ophthalmology.

[23] He N., Wu L., Qi M., He M., Lin S., Wang X., Yang F., Fan X. (2016). Comparison of Ciliary Body Anatomy between American Caucasians and Ethnic Chinese Using Ultrasound Biomicroscopy. Curr. Eye Res..

[24] Okamoto Y., Okamoto F., Nakano S., Oshika T. (2017). Morphometric assessment of normal human ciliary body using ultrasound biomicroscopy. Graefe’s Arch. Clin. Exp. Ophthalmol..

[25] Li J., Drechsler J., Lin A., Widlus M., Qureshi A., Stoleru G., Saeedi O., Levin M.R., Kaleem M., Jaafar M. (2021). Repeatability and Reliability of Quantified Ultrasound Biomicroscopy Image Analysis of the Ciliary Body at the Pars Plicata. Ultrasound Med. Biol..

[26] Buckhurst H., Gilmartin B., Cubbidge R.P., Nagra M., Logan N.S. (2013). Ocular biometric correlates of ciliary muscle thickness in human myopia. Ophthalmic Physiol. Opt..

[27] Fernández-Vigo J.I., Shi H., Kudsieh B., Arriola-Villalobos P., De-Pablo Gómez-de-Liaño L., García-Feijóo J., Fernández-Vigo J. (2020). Ciliary muscle dimensions by swept-source optical coherence tomography and correlation study in a large population. Acta Ophthalmol..

[28] Khodeiry M.M., Sheheitli H., Sayed M.S., Persad P.J., Feuer W.J., Lee R.K. (2021). Treatment Outcomes of Slow Coagulation Transscleral Cyclophotocoagulation In Pseudophakic Patients with Medically Uncontrolled Glaucoma. Am. J. Ophthalmol..

[29] Tóth M., Shah A., Hu K., Bunce C., Gazzard G. (2019). Endoscopic cyclophotocoagulation (ECP) for open angle glaucoma and primary angle closure. Cochrane Database Syst. Rev..

[30] Giannaccare G., Pellegrini M., Bernabei F., Urbini L., Bergamini F., Ferro Desideri L., Bagnis A., Biagini F., Cassottana P., Del Noce C. (2021). A 2-year prospective multicenter study of ultrasound cyclo plasty for glaucoma. Sci. Rep..

